# Solitary fibrous tumor in the liver: case report and literature review

**DOI:** 10.1186/s40792-019-0625-6

**Published:** 2019-04-24

**Authors:** Kyohei Yugawa, Tomoharu Yoshizumi, Yohei Mano, Takeshi Kurihara, Shohei Yoshiya, Kazuki Takeishi, Shinji Itoh, Noboru Harada, Toru Ikegami, Yuji Soejima, Kenichi Kohashi, Yoshinao Oda, Masaki Mori

**Affiliations:** 1 Department of Surgery and Science, Graduate School of Medical Sciences, Kyushu University, Fukuoka, 812-8582 Japan; 2 Department of Anatomic Pathology, Graduate School of Medical Sciences, Kyushu University, Fukuoka, Japan

**Keywords:** Solitary fibrous tumor, Malignant, Radiology and pathology

## Abstract

**Background:**

Solitary fibrous tumors (SFTs) are uncommon mesenchymal neoplasms that present most commonly at intrathoracic sites. SFTs of the liver are rare, with only a few having been reported in the English-language literature. We report a rare case of a hepatic SFT and literature review.

**Case presentation:**

A 49-year-old woman underwent surgery for a cranial hemangiopericytoma two decades previously. She currently presented with malaise. Abdominal computed tomography (CT) showed a huge, sharply demarcated mass in the anterior segment of the liver. Tumor marker levels were within the normal range. Following central bisegmentectomy of the liver, histological examination of the specimen revealed that the tumor was composed of spindle and fibroblast-like cells with collagenous stroma. Immunohistochemically, the spindle cells were negative for CD34 but positive for STAT6. The *NAB2–STAT6* fusion gene was detected by the reverse transcription polymerase chain reaction. A diagnosis of SFT was thus confirmed histopathologically and genetically.

**Conclusions:**

The SFT of the liver is an uncommon finding. Because there are no specific imaging features, it is difficult to diagnose the hepatic SFT preoperatively. We consider that careful surgical resection and postoperative follow-up are necessary for hepatic SFTs.

## Background

Solitary fibrous tumors (SFTs) of the liver are uncommon benign tumors. The disease is defined by the World Health Organization (WHO) as a benign tumor originating from submesothelial tissue. Its histological features include bland, uniform, fibroblast-like spindle cells and branching hemangiopericytoma-like vessels [1]. Rarely, it is found at extra-thoracic sites, including the mediastinum, skin, meninges, orbit, upper respiratory tract, breast, thyroid, and peritoneum. The hepatic SFT is extremely rare. To our knowledge, including our patient, only 85 cases have been reported in the English-language literature. In addition, there have been few clinicopathological studies of this rare disease, and the imaging features are non-specific, making an imaging diagnosis difficult. Because of this difficulty, the definitive diagnosis is typically based on histopathological and immunohistochemical features [2, 3].

Immunohistochemically, the cluster of differentiation 34 (CD34) is a positive marker for mesenchymal tumor cells such as are found in SFTs, epithelioid sarcomas, and gastrointestinal stromal tumors (GISTs), exhibiting undesirable imperfections in sensitivity and specificity. In fact, 5–10% of SFTs are nonreactive to CD34, making the differential diagnosis of SFTs based on histopathology difficult [4]. Recently, several studies revealed outstanding progress in the diagnosis of SFT based on a fusion gene of juxtaposed NGFI-A binding protein 2 (NAB2) and signal transducer and activator of transcription 6 (STAT6) using both whole-exome sequencing and integrative sequencing [4–6]. STAT6 has been identified as a highly sensitive, almost completely specific immunohistochemical marker, which distinguishes it from its mimics [7–9]. Although most SFTs exhibit benign behavior, some have malignant features, including local recurrence [10]. We present a rare case of SFT of the liver and discuss its radiological and pathological diagnostic features, as well as the possibility of malignancy.

## Case presentation

A 49-year-old woman had a history of treatment for a cranial meningioma that was diagnosed histologically as a hemangiopericytoma two decades previously. Currently, she had no co-morbidities and no alcohol abuse, and she was negative for hepatitis B/C virus. She presented with a 1-month history of malaise of unknown cause and abdominal bloating. Analysis of serum tumor markers revealed none that were elevated, including α-fetoprotein (3.5 ng/ml), protein induced by vitamin K absence or antagonist-2 (21 mAU/ml), carbohydrase antigen 19–9 (19.2 U/ml), and carcinoembryonic antigen (0.8 ng/ml). Other parameters were within their normal ranges.

**Fig. 1:**
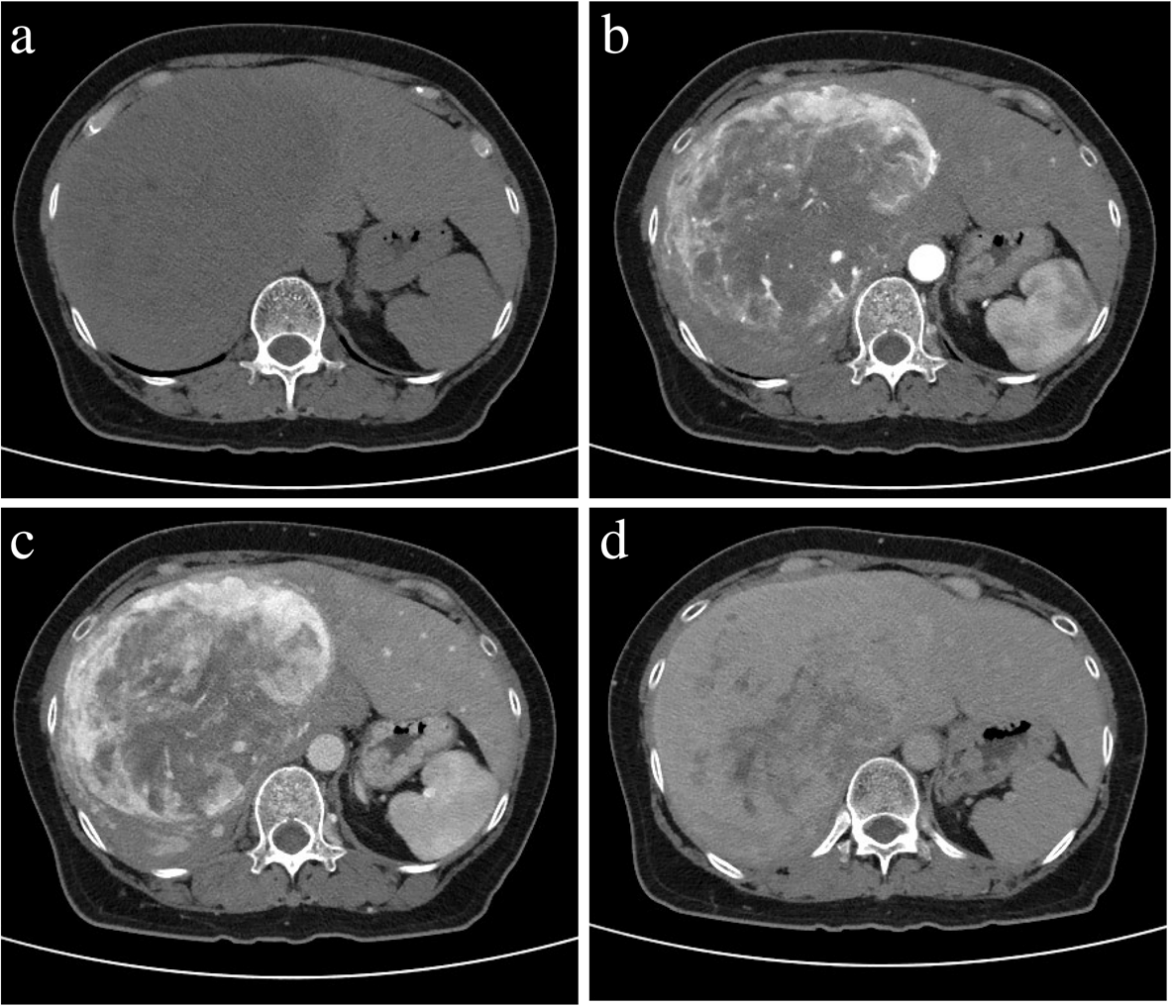
Contrast-enhanced abdominal computed tomography (CT). Plain CT shows a well-defined low-density mass occupying the right lobe (14.0 cm diameter) (**a**). Contrast-enhanced CT shows heterogeneous enhancement in the peripheral mass during the arterial phase (**b**). Enhancement is then centripetal and more pronounced during the portal phase (**c**) and finally progresses to persistent, less heterogeneous enhancement during the delayed phase (**d**)

The patient underwent abdominal computed tomography (CT), which revealed a large mass involving almost the entire right lobe of the liver. It measured 14 cm in maximum diameter and was compressing the inferior vena cava (Fig. 1a). Contrast-enhanced CT showed marked heterogeneous enhancement in the periphery of the mass during the arterial phase (Fig. 1b), with the enhancement becoming centripetal and more pronounced in round unenhanced areas related to necrotic or cystic changes during the portal phase (Fig. 1c). It finally progressed to persistent, less heterogeneous enhancement during the delayed phase (Fig. 1d).

**Fig. 2:**
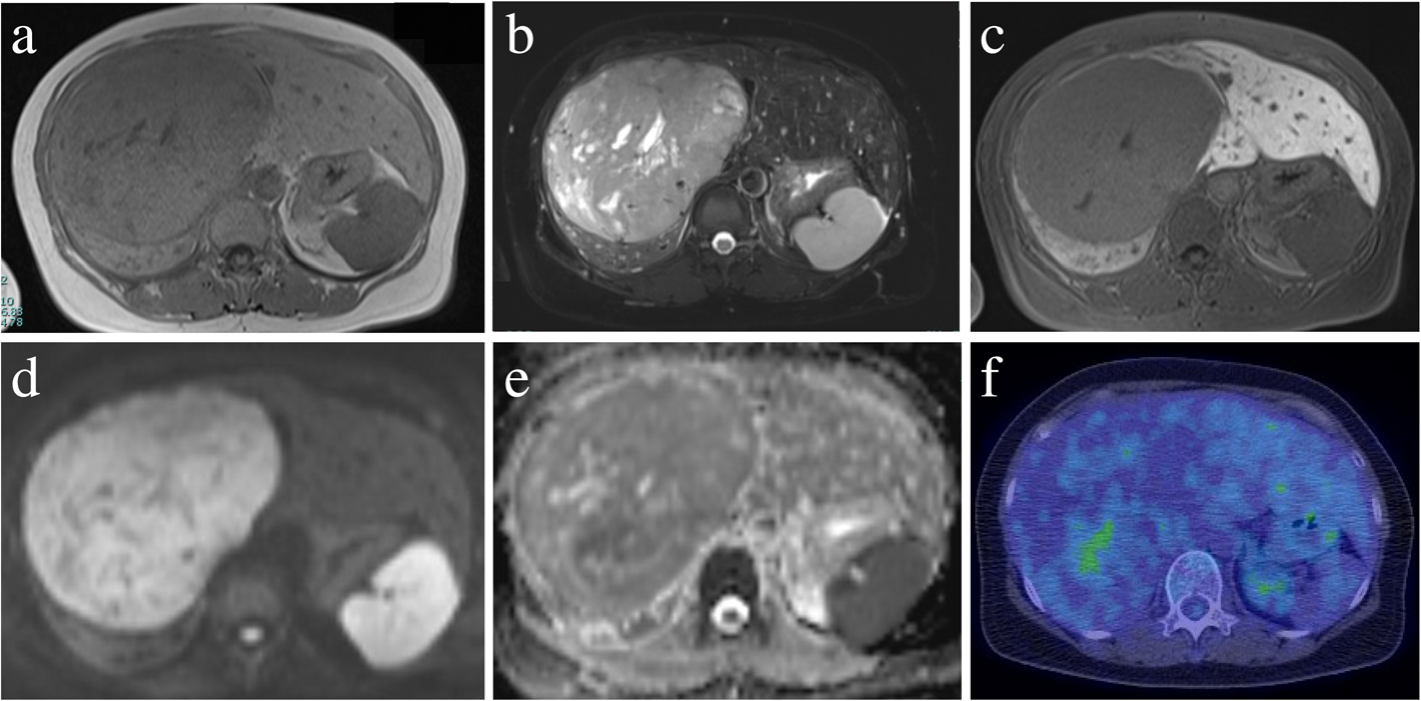
Magnetic resonance imaging (MRI) and [^18^F]-fluorodeoxyglucose positron emission tomography (FDG-PET). Abdominal MRI shows low intensity on a T1-weighted image (**a**) and heterogeneously high or iso intensity on a T2-weighted image (**b**). Gadolinium-ethoxybenzyl-diethylenetriamine pentaacetic acid-enhanced MRI reveals a hypointense mass during the hepatobiliary phase (**c**). Diffusion-weighted imaging shows higher intensity than for normal liver parenchyma (**d**) with a high *b* value of 1000 (**e**). FDG-PET shows no accumulation of [^18^F]-FDG (**f**)

Abdominal magnetic response imaging (MRI) showed low intensity on T1-weighted images and heterogeneously high or iso intensity on T2-weighted images (Fig. 2a, b). Furthermore, it showed higher intensity than that of normal liver parenchyma on diffusion-weighted imaging (DWI) with a high *b* value of 1000 (Fig. 2d, e). Gadolinium-ethoxybenzyl-diethylenetriamine pentaacetic acid-enhanced magnetic response imaging (EOB-MRI) revealed a hypointense mass during the hepatobiliary phase (Fig. 2c). [^18^F]-fluorodeoxyglucose-positron emission tomography (FDG-PET) showed no accumulation of [^18^F]-FDG (Fig. 2f). Radiological evaluation found nothing to suggest the presence of a tumor mass anywhere in the body, including no cranial or spinal lesions. Gastroscopy and colonoscopy findings were normal. According to radiological examination, the preoperative diagnosis was a malignant tumor, such as scirrhous hepatocellular carcinoma (HCC), sarcomatous HCC, GIST, or hemangiosarcoma. The patient then underwent central bisegmentectomy of the liver.

**Fig. 3:**
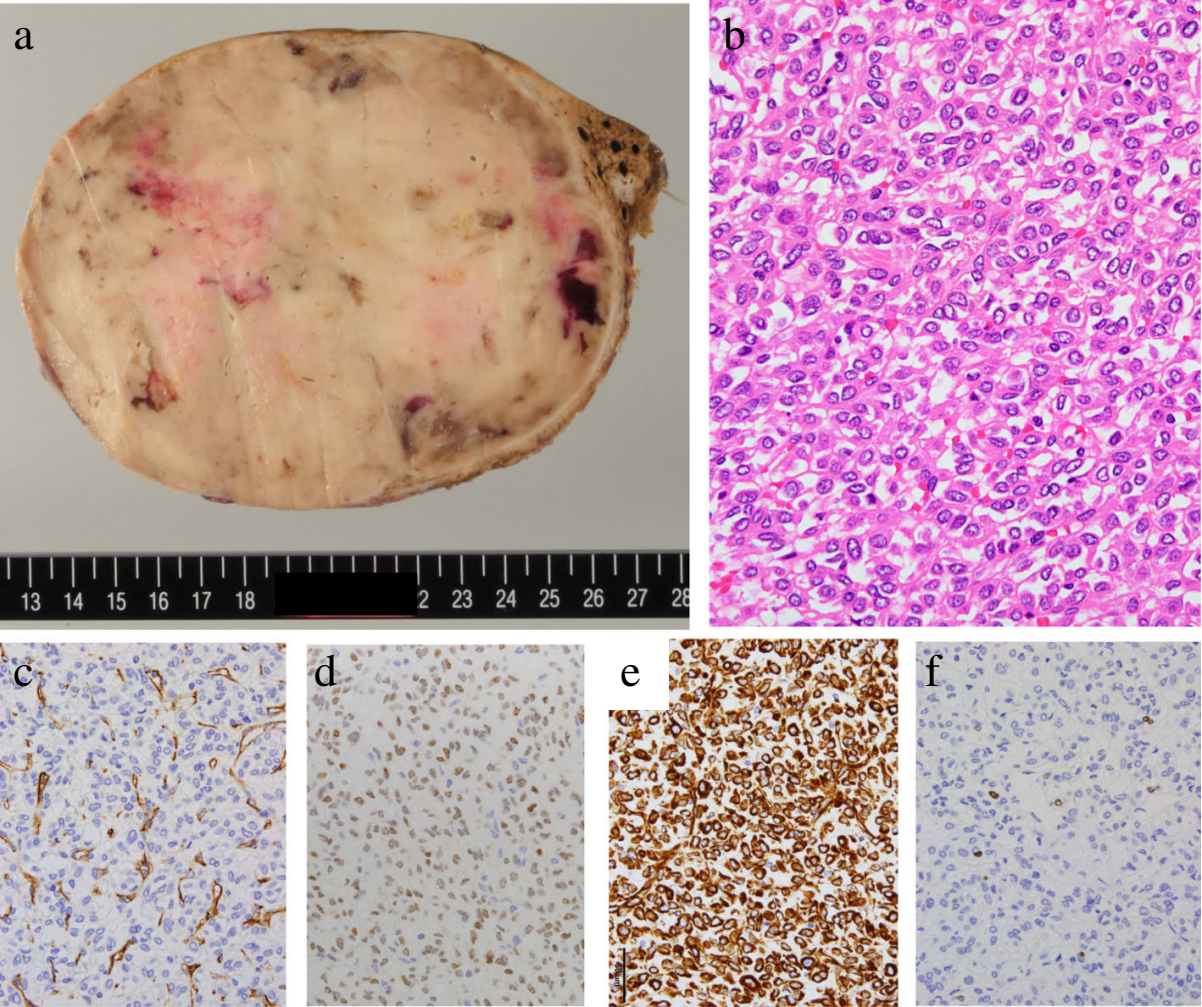
Macroscopic and microscopic findings of SFT. Macroscopically, the tumor mass was firm and yellowish-white with an intact capsule (13.3 cm maximum diameter) (**a**). Microscopically, the tumorous tissue showed a proliferation of oval to short spindle-shaped cells arranged in a “pattern-less pattern” (H&E × 400) (**b**). Immunohistochemically, the tumor cells were negative for CD34 (× 400; **c**), but positive for STAT6 (nuclei, × 400) (**d**) and vimentin (× 400) (**e**). Ki67 labeling index was < 5% (× 400) (**f**)

Macroscopically, the maximum diameter of the tumor was 13.3 cm, and the tumor itself was firm and yellowish-white with an intact capsule. Hemorrhagic areas and horizontal intertwined fiber bundles were observed on the cut surface of the tumor (Fig. 3a). Microscopically, the tumorous tissue showed proliferation of oval to short spindle-shaped cells arranged in no particular pattern, accompanied by focal fibro-collagenous or myxoid stroma and a few hemangiopericytomatous branching vessels. Foci of hemorrhage and necrosis were observed. Mitotic figures were present, although rare, highlighted by hematoxylin and eosin (HE) staining [< 1/20 high-power fields (HPFs)] (Fig. 3b). There was no vascular or parenchymal invasion.

**Fig. 4:**
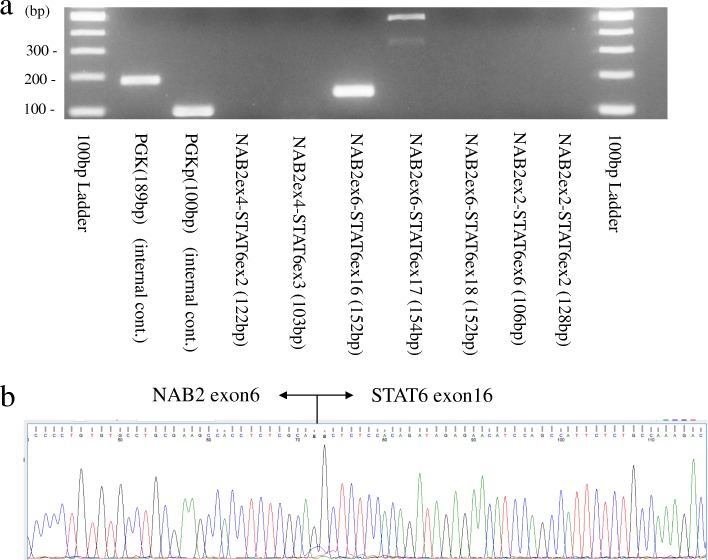
*NAB2–STAT6* fusion gene in the SFT identified by RT-PCR and sequencing. Gel electrophoresis of PCR products reveals the various *NAB2–STAT6* fusion genes with heterogeneous exon compositions in the tumor, which were identified using seven primer pairs (**a**). Direct sequencing shows the junction breakpoint in a stretch of *NAB2* intronic sequence between the 3′-end of the NAB2 exon 6 and the 5′-end of the STAT6 exon 16 (**b**)

Immunohistochemically, the tumor cells were negative for CD34 (Fig. 3c) but positive for STAT6 (Fig. 3d) and vimentin (Fig. 3e). No markers of HCC (hepatocytes, glypican-3) or GIST (S100 protein, cKIT, DOG1) were conspicuous in the specimen (not shown in Fig. 3). The Ki67 labeling index was < 5% (Fig. 3f). *NAB2–STAT6* fusion gene was detected by reverse transcription-polymerase chain reaction (RT-PCR) and direct sequencing. Gel electrophoresis of PCR products identified various *NAB2–STAT6* fusions with heterogeneous exon compositions in the tumor using seven primer pairs (Fig. 4a). Direct sequencing showed the junction breakpoint in a stretch of NAB2 intronic sequences between the 3′-end of NAB2 exon 6 and the 5′-end of STAT6 exon 16 (Fig. 4b). Thus, SFT of the liver was diagnosed definitively based on these histological and genetic results. The surrounding liver showed mild inflammation in the normal portal area. Following surgery, the patient recovered uneventfully. At present, 12 months postoperatively, she remains well with no evidence of tumor recurrence.

## Discussion

First described in 1931 [11], SFT has since appeared in the form of hemangiopericytoma, giant cell angiofibroma, fat-forming variant, lipomatous hemangiopericytoma, and, rarely, as a mesenchymal neoplasm that commonly originates from pleura. In the 2013 WHO classification of tumors of soft tissue and bone, extrapleural SFT was considered a fibroblastic/myofibroblastic neoplasm with intermediate, rarely metastasizing biological behavior [1]. Subsequently, in the updated WHO classification of the digestive system, SFT is considered a benign tumor with the potential for malignant transformation [12]. Because the hepatic SFT is extremely rare, whether the SFT should be classified as a benign or malignant tumor remains controversial regarding this tumor originating from the liver.

Only 85 patients with SFTs of the liver, including ours, have been reported in the English-language literature. According to the review by Chen et al. [13], the average age of patients with this affliction is 57.1 (range 16–87) years. It appears to occur more frequently in women than in men (1.4:1.0), and the histological incidence of malignant features is 19.0% (16/84 patients). The clinical presentation of this disease is generally non-specific. Extrapleural SFTs present as relatively slow-growing masses that are often asymptomatic. When symptoms do occur, they are caused by the pressure the mass exerts on adjacent structures [14]. Similarly, our patient complained of steadily increasing abdominal bloating at presentation, which proved to be due to the huge tumor compressing other organs. In previous reports, hepatic SFTs were found incidentally during routine examinations.

Among radiological studies, ultrasonography often reveals a heterogeneous mass that may be either hypoechogenic or hyperechogenic (or both) with or without calcification. Contrast-enhanced CT shows early arterial enhancement with delayed venous washout. MRI of our patient showed that the tumor mass was heterogeneous with slight hyperintensity on T2-weighted images and slight hypointensity on T1-weighted images. Findings on MRI are similar to those seen on CT scans. DWI revealed greater signal intensity than that of normal liver [15]. These findings often mimic those of high-grade HCCs (including scirrhous and sarcomatous HCC) or leiomyomas. Our radiological results were consistent with these patterns.

In our case, a percutaneous biopsy was not performed to obtain a tissue diagnosis. Although a fine-needle biopsy can distinguish the SFT from malignant tumors such as HCC or sarcoma, the procedure could lead to rupture or seeding of malignant cells. Some reports suggested that, although liver biopsy could be performed safely, the biopsy of the SFT was misdiagnosed as HCC or metastasis from adenocarcinoma of the pancreas or a gastrointestinal tract lesion [16, 17]. In our patient, the possibility of a malignant tumor could not be ruled out preoperatively because we decided to resect this huge tumor without a definitive diagnosis due to the patient’s symptoms and the risk of hemorrhage.

A definitive diagnosis of SFT of the liver requires histopathological and immunohistochemical studies. Microscopically, the tumor is composed of ovoid spindle-shaped cells with its characteristic architecture in a storiform pattern or a haphazard, “patternless pattern.” These cells are separated from thick bands of keloid-like collagen bundles and display branching of staghorn vessels in a hemangiopericytoma-like pattern. Myxoid changes are also commonly observed. Mitoses and necrotic changes—characteristically suggesting malignancy—are rare for this tumor [13]. Features identified by WHO as being associated with malignancy include hypercellularity, cytological atypia, tumor necrosis, infiltrative margins, and high mitotic activity (≥ 4/10 HPF) [1].

Immunohistochemically, the staining of CD34, vimentin, and Bcl-2 is useful for distinguishing SFTs from other liver tumors. However, CD34 staining is imprecise in this case because 5–10% of typical SFTs are nonreactive to CD34 [1]. To make up for this imprecision, the *NAB2–STAT6* fusion gene has recently been identified as the genetic hallmark of SFT. This aberration drives the nuclear relocation of STAT6 [6]. Immunohistochemical detection of STAT6 nuclear expression and the *NAB2–STAT6* fusion gene identified by RT-PCR assay offers a strong surrogate diagnostic technique for distinguishing SFTs from histological mimics. Our patient, in fact, was immunohistochemically negative for CD34, which made the definitive diagnosis difficult. However, the spindle cells were immunohistochemically positive for STAT6, and the *NAB2–STAT6* fusion gene was detected by RT-PCR and direct sequencing, thereby allowing a definitive SFT diagnosis.

Chen et al. [13] previously reported that 16 of 84 SFTs were malignant, which was similar to the intrapleural SFT recurrence rate of 20–67% among malignant tumors. These malignant cases were diagnosed by histological examination, which showed a high incidence of mitotic changes (> 4/10 HPF) and local recurrence or distant metastasis in 17.9%. Furthermore, 26.7% of patients with these malignant SFTs had a local recurrence within 9 months to 6 years, and 53% had distant metastasis within 1 month to 6 years. England et al. [18] established the criteria for malignant SFT: mitotic changes (> 4/10 HPFs), tumor necrosis and hemorrhage, nuclear pleomorphism, and metastasis were the major criteria, and large tumor size (> 10 cm) and cellular atypia were the minor criteria. Wilky et al. [19] classified the SFTs into “benign” with no England’s criteria, “borderline” with 1 or more England’s criteria but final classification as benign, and “malignant.” This report described that “borderline” SFTs with any of England’s criteria had been related to high risk of recurrence. Our patient described herein met two of the six criteria (necrosis/hemorrhage and tumor size), suggesting a possibility of malignancy.

SFTs of the head, neck, and intracranial meninges are also rare and display benign behavior without metastasis [20]. We found no descriptions in the English-language literature that resembled that of our patient, who presented with suspected localized liver metastasis from a head SFT. Du et al. [21], however, reported a rare case of non-malignant SFT that appeared 5 years after initial liver resection. The tumor’s appearance had no marked variances from other non-malignant SFTs.

To clarify whether this second tumor was a recurrence from a cranial SFT in our patient, we attempted to examine specimens from her previous, cranial SFT. Unfortunately, no tissue was available because the maintenance term of the specimen had reached its limit, and it had been discarded. Assessment of the radiological findings, however, showed that there had been no abnormality in other organs, and no important mitotic changes or low levels of the Ki67 labeling index. Thus, it was more likely that our patient currently had a primary tumor in the liver rather than a recurrence from her original cranial SFT. Nevertheless, we are committed to performing carefully follow-up every 3 months in the first 2 years, twice each year up to 5 years after surgery, and then once a year after the fifth year. The best method for follow-up is not well established, but whole-body CT is suggested in our patient.

## Conclusions

According to previous reports, SFT of the liver is an extremely rare and benign tumor. Because of the few clinicopathological specific features, it is difficult to diagnose this tumor. We performed the resection of the tumor and definitively diagnosed SFT of the liver histopathologically and genetically. We consider that careful surgical resection and postoperative follow-up are necessary for hepatic SFTs.

## Abbreviations

CD34: Cluster of differentiation 34
CT: Computed tomography
DWI: Diffusion-weighted imaging
EOB-MRI: Gadolinium-ethoxybenzyl-diethylenetriamine pentaacetic acid-enhanced magnetic response imaging
FDG-PET: [^18^F]-fluorodeoxyglucose positron emission tomography
GIST: Gastrointestinal stromal tumor
HCC: Hepatocellular carcinoma
HE: Hematoxylin and eosin
MRI: Magnetic response imaging
NAB2: NGFI-A binding protein 2
RT-PCR: Reverse transcription polymerase chain reaction
SFT: Solitary fibrous tumor
STAT6: Signal transducer and activator of transcription 6
WHO: World Health Organization
